# Radiographic Healing After Intramedullary Nailing with or Without Lateral Plate Augmentation in Atypical Subtrochanteric Femoral Fractures: A Retrospective Study

**DOI:** 10.3390/jcm14144976

**Published:** 2025-07-14

**Authors:** Le Wan, Chan-Young Lee, Taek-Rim Yoon, Kyung-Soon Park

**Affiliations:** Department of Orthopedic Surgery, Chonnam National University Medical School and Hwasun Hospital, Hwasun 58128, Republic of Korea; wle202302@gmail.com (L.W.);

**Keywords:** hip fractures, intramedullary nailing, bone plates, fracture healing, radiographic image interpretation

## Abstract

**Background**: Atypical subtrochanteric femoral fractures (ASFs), frequently linked to long-term bisphosphonate use, present significant fixation challenges due to impaired bone healing. While intramedullary (IM) nailing is the standard treatment, delayed union or nonunion remains common. This study aimed to evaluate whether supplementing IM nailing with lateral plate augmentation improves radiographic healing in patients with ASFs. **Methods**: This retrospective comparative study included 12 elderly female patients with ASFs treated between October 2013 and October 2023. Five patients underwent IM nailing alone (IM group), while seven received IM nailing with additional lateral plate fixation (Plate + IM group). Fracture healing was assessed using the modified Radiographic Union Score for Tibial fractures (mRUST) at 3, 6, and 12 months postoperatively. Intergroup comparisons were performed using the Mann–Whitney U test. **Results**: The median mRUST scores in the IM group were 4 (IQR 3.5–4), 6 (IQR 4.5–6.5), and 8 (IQR 7–9) at 3, 6, and 12 months, respectively. In the Plate + IM group, the scores were 5 (IQR 4–6), 8 (IQR 8–8), and 10 (IQR 10–11), respectively. The Plate + IM group demonstrated significantly higher mRUST scores at all assessed time points (3 months: *p* = 0.018; 6 months: *p* = 0.003; 12 months: *p* = 0.006). No implant failures or postoperative infections occurred in either group during the 12-month follow-up period. One patient (20%) in the IM group developed fracture nonunion, while no nonunion cases were observed in the Plate + IM group. **Conclusions**: Lateral plate augmentation as an adjunct to IM nailing may promote faster and more consistent radiographic healing in atypical subtrochanteric femoral fractures. This dual-fixation strategy may offer a biomechanically more robust option for patients at risk of delayed union, potentially contributing to a lower risk of nonunion, though further prospective studies are required to confirm this finding.

## 1. Introduction

Atypical subtrochanteric femoral fractures (ASFs), defined as fractures occurring within 5 cm distal to the lesser trochanter, represent a rare but challenging subset of femoral fractures, accounting for approximately 2.95% of all femoral fractures [1]. The annual incidence ranges from 3 to 9.8 per 100,000 individuals and is particularly elevated among postmenopausal Asian women. These fractures are commonly associated with low-energy trauma and long-term bisphosphonate use, which are factors that impair bone turnover and disrupt normal biomechanics, often leading to suppressed bone remodeling and increased microdamage accumulation [2,3,4].

The healing of ASFs is notoriously difficult due to the unique anatomical and biomechanical characteristics of the subtrochanteric region. This critical area is subjected to complex and high-stress forces, including significant medial compressive forces and lateral tensile forces, which can lead to persistent micromotion at the fracture site and inhibit callus formation. Furthermore, the subtrochanteric region often exhibits limited cortical blood supply, which compromises the biological environment necessary for robust bone healing. The wide medullary canal in this proximal femoral segment also presents a biomechanical challenge, as it often prevents close cortical contact with standard intramedullary nails, thereby reducing construct stability and predisposing to delayed union or nonunion. These combined factors make ASFs inherently prone to impaired and protracted healing. Several studies have reported healing times exceeding 6–8 months [5], with subtrochanteric fractures healing more slowly than diaphyseal atypical femoral fractures [3,6,7].

Long cephalomedullary nailing (IM nail) remains the standard treatment for ASFs due to its minimally invasive nature and ability to bear axial load [6,8]. However, despite this approach, nonunion rates remain notably high, ranging from 7% to 20%, a rate substantially greater than those observed in typical femoral fractures [9,10,11]. This elevated nonunion risk is primarily attributed to the anatomical challenges of the subtrochanteric region; the wide medullary canal often prevents close cortical contact with the nail, resulting in suboptimal stability, particularly against torsional and varus stresses. Such compromised biomechanical stability frequently leads to delayed healing. Consequently, many patients with IM nailing alone may require secondary procedures to address nonunion, which significantly increases both morbidity and healthcare costs [12].

While closed reduction techniques often employed with IM nailing aim to minimize soft tissue disruption, achieving precise anatomical reduction can be challenging in comminuted or significantly displaced ASFs due to the powerful muscle forces acting on the fragments. Conversely, open reduction, typically performed in conjunction with plate augmentation, offers the advantage of direct visualization and facilitates more accurate anatomical alignment, which is often crucial for achieving optimal fracture healing and restoring limb mechanics. However, it is important to acknowledge that open reduction can potentially lead to a greater sterile inflammatory response and soft tissue trauma compared to minimally invasive approaches, a factor that requires careful consideration in fracture management, particularly in elderly patients with compromised healing capacity [13].

**Figure 1:**
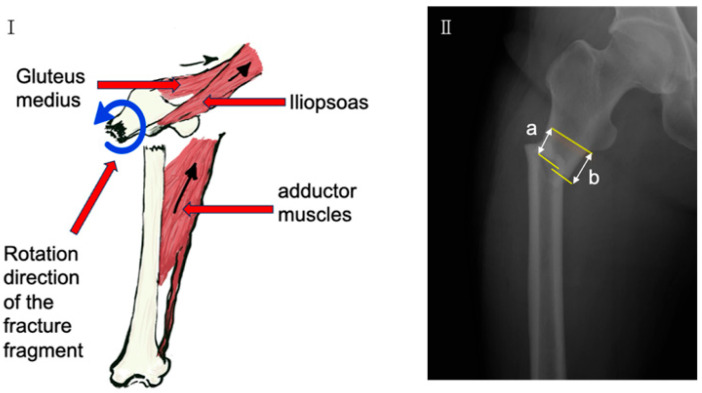
Illustration of atypical subtrochanteric femoral fracture (ASF) morphology and relationship to lesser trochanter. (**I**) Schematic depicting typical displacement patterns in ASFs influenced by muscle forces relevant for surgical planning, Arrows indicate cortical gaps and the rotational direction of the distal fracture fragment, Black arrows indicate the direction of muscle pull. (**II**) Radiographic view of an ASF highlighting (a) the vertical distance of the lateral bone cortex at the fracture end from the lesser trochanter and (b) the distance of the medial bone cortex at the fracture end from the lesser trochanter used in preoperative assessment.

To enhance fixation stability and promote union, lateral plate augmentation has been proposed as an adjunct to IM nailing. A lateral plate can provide a stable buttress, assisting with anatomical reduction—particularly in displaced fractures influenced by muscle forces (Figure 1I)—and significantly improving the overall rigidity and torsional stability of the osteosynthesis construct [14]. While favorable outcomes have been reported using plates for ASF nonunion after failed IM nail fixation [9,12,15], it is crucial to note that such cases typically involve secondary salvage procedures, necessitating revision surgery, which carries additional risks and costs.

In contrast to these established salvage approaches for failed IM nailing, a key distinguishing feature and novelty of our study is its focus on evaluating the efficacy of primary plate-augmented IM nailing during the initial surgical intervention for ASFs. Given the inherent challenges of ASF healing and the limitations of IM nailing alone, there is a growing interest in exploring the efficacy of such a primary dual-fixation strategy during initial surgery. This study, therefore, aimed to evaluate radiographic healing and key treatment-related outcomes (such as nonunion- and implant-related complications) in patients with ASFs treated with IM nailing alone versus IM nailing combined with lateral plating. By investigating whether this enhanced dual-fixation strategy could accelerate healing, potentially reduce the risk of nonunion, and optimize the initial management of these complex fractures, we sought to provide preliminary insights for a more robust primary surgical approach.

## 2. Materials and Methods

### 2.1. Study Design and Patient Selection

This retrospective comparative study was approved by the Institutional Review Board of Chonnam National University Hwasun Hospital (IRB No. CNUHH-2025-060). The aim was to evaluate the efficacy of combining lateral plate augmentation with intramedullary (IM) nailing for the treatment of atypical subtrochanteric femoral fractures (ASFs).

Between October 2013 and October 2023, 12 elderly female patients diagnosed with ASF at Chonnam National University Hospital were included. Diagnosis was based on the criteria outlined in the second report by the Task Force of the American Society for Bone and Mineral Research [16]. Patients included in this retrospective study were categorized into two treatment groups based on the surgical fixation method used for their atypical subtrochanteric femoral fracture treated between October 2013 and October 2023. The IM group (*n* = 5) consisted of patients treated with cephalomedullary nailing (CMN) (Zimmer, Warsaw, IN, USA) alone, predominantly from the earlier part of the study period. The Plate + IM group (*n* = 7) comprised patients who received CMN with additional lateral plate augmentation, a technique that was introduced and increasingly adopted in our department for these fractures from approximately January 2016 onwards. Retrospective analysis indicated that preoperative and intraoperative baseline characteristics were comparable between the two groups (Table 1).

Inclusion criteria:

- Female patients aged ≥60 years;
- Surgical treatment performed within one week of injury;
- Minimum of 12 months of clinical and radiographic follow-up.

Exclusion criteria:

- Prior surgery on the ipsilateral femur;
- Pathological fractures unrelated to bisphosphonate use or osteoporosis;
- Severe medical comorbidities precluding surgery;
- Incomplete follow-up or missing radiographic data.

All patients in both groups received CMN femoral nails (Zimmer, Warsaw, IN, USA). Patients in the Plate + IM group additionally received a narrow locking compression plate (LCP; DePuy Synthes, Raynham, MA, USA) secured using two cables and sleeves (Hankil Co., Ltd., Gimpo-si, Gyeonggi-do, Republic of Korea).

### 2.2. Surgical Procedure

All surgeries were performed under fluoroscopic guidance by senior orthopedic surgeons.

IM Group: Patients were placed in the supine position on a radiolucent table with a support pad under the affected hip. Following closed reduction under C-arm guidance, a long femoral intramedullary nail was inserted. Proximal fixation was achieved using a lag screw into the femoral neck, and two distal locking screws were inserted to secure the construct.

Plate + IM Group: Patients allocated to the Plate + IM group were consistently positioned in the lateral decubitus position on a standard radiolucent operating table. A direct lateral approach was employed; an incision was made through the skin and subcutaneous tissue, followed by an incision of the tensor fasciae lata in line with its fibers to expose the vastus lateralis muscle, which was then split or retracted anteriorly to reveal the anterolateral surface of the femur and the subtrochanteric fracture site. The fracture site was meticulously exposed, ensuring minimal soft tissue stripping to preserve vascularity. Anatomical reduction of the fracture fragments was then achieved under direct visualization, aided by manual traction, K-wires, or reduction clamps as necessary.

**Figure 2:**
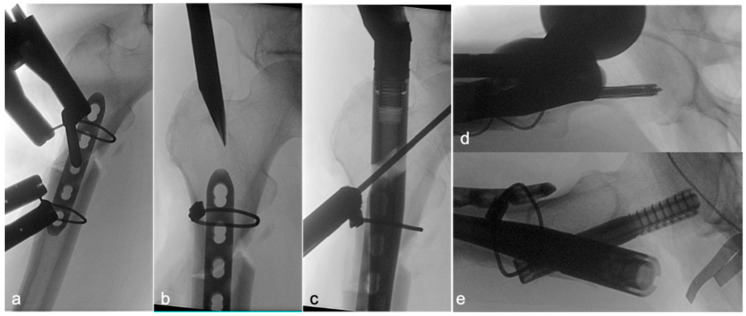
Key intraoperative C-arm images illustrating the surgical sequence of intramedullary (IM) nailing with lateral plate augmentation for an atypical subtrochanteric femoral fracture. (**a**) (AP) The contoured lateral plate is secured to the femur using two Dall–Miles cable systems. (**b**) (AP) Following initial fracture reduction and plate fixation, the guide pin for the intramedullary nail is inserted into the proximal femur. (**c**) (AP) Targeting for the cephalic/lag screw of the intramedullary nail. (**d**) Placement and confirmation of the cephalic/lag screw (lateral). (**e**) The completed dual-fixation construct, showing the intramedullary nail with proximal locking, and the augmentative lateral plate in situ, confirming satisfactory fracture alignment and implant positioning (lateral).

**Figure 3:**
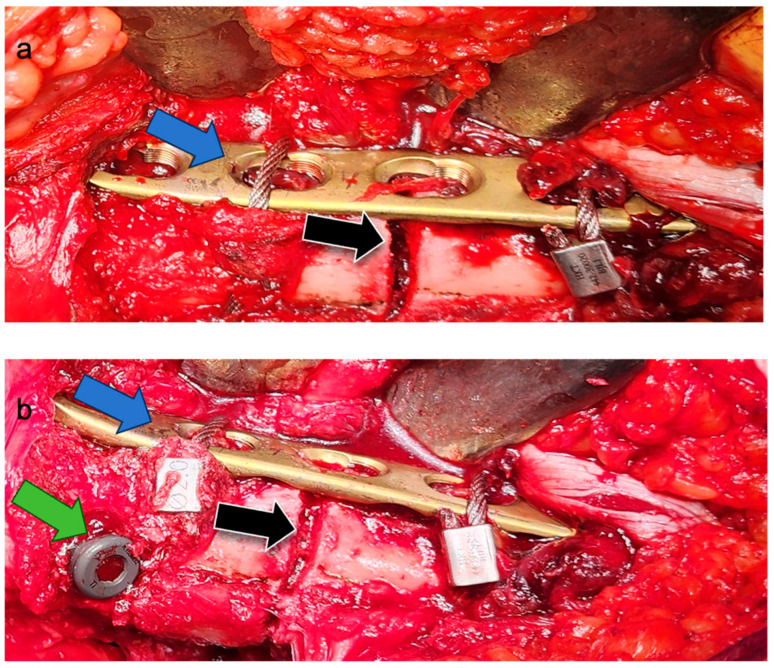
Intraoperative clinical photographs showing the fracture site and implant construct during surgical fixation in the Plate + IM group for an atypical subtrochanteric femoral fracture. (**a**) Clinical view after anatomical reduction and definitive fixation of the contoured lateral plate with Dall–Miles cables prior to intramedullary nail insertion. The fracture line (black arrow) and lateral plate (blue arrow) are clearly visible. (**b**) Final construct after intramedullary nail insertion with proximal and distal locking screws. The head screw of the nail (green arrow), the lateral plate (blue arrow), and the fracture site (black arrow) are demonstrated prior to wound closure.

Following satisfactory reduction, a 4–5-hole narrow locking compression plate (LCP; DePuy Synthes; Raynham, MA, USA) was carefully contoured to conform to the anterolateral aspect of the proximal femur, spanning the fracture. The plate was provisionally stabilized and subsequently secured definitively to the femur using two Dall–Miles cable systems (Hankil Co., Ltd.; Gimpo-si, Gyeonggi-do, Republic of Korea). The placement and security of the plate were confirmed by intraoperative C-arm imaging (Figure 2a) and direct clinical inspection of the implant–bone interface at the fracture site (Figure 3a).

Once the lateral plate was securely fixed, providing augmented stability and maintaining the reduction, a guide pin for the cephalomedullary nail (CMN) (Zimmer; Warsaw, IN, USA) was inserted through the standard trochanteric entry point under fluoroscopic guidance (Figure 2b). The medullary canal was then reamed according to standard technique. Subsequently, the CMN was carefully inserted across the fracture site, passing alongside the prepositioned augmentative plate. The proximal fixation of the nail was achieved by inserting a cephalic/lag screw into the femoral head and neck, with its trajectory and depth confirmed by fluoroscopy (Figure 2c,d). Finally, two distal interlocking screws were placed through the nail to secure the distal fixation. The completed dual-fixation construct, ensuring satisfactory fracture alignment, implant stability, and overall mechanical integrity, was verified with final C-arm images (Figure 2e) and direct intraoperative visualization of the stabilized fracture site prior to wound closure (Figure 3). Postoperative rehabilitation protocols were standardized for both groups, with weight-bearing restrictions guided by radiographic evidence of healing and surgeon discretion.

### 2.3. Data Collection

Clinical and radiographic data were retrospectively collected from electronic medical records and the picture archiving and communication system (PACS) from the time of surgery through a minimum of 12 months of follow-up. Data included demographic information, operative records, radiographic images, and notes on basic ambulatory status where available. Radiographic healing was assessed using the modified Radiographic Union Score for Tibial fractures (mRUST) at 3, 6, and 12 months postoperatively [17,18,19].

The following variables were analyzed and compared between groups: age, weight, height, body mass index (BMI), bone mineral density (BMD) of the hip and spine, lateral and medial cortical gaps (Figure 1II), pre- and postoperative hemoglobin levels, hemoglobin drop, estimated blood loss, operative time, intramedullary nail diameter, and the canal-to-nail diameter difference [20].

The blood loss estimates were based on the literature [21]. Blood volume (BV) was estimated on the basis of Gross’s formula BV = 65 × weight (kg).

$$\begin{array}{r} \mathrm{Calculated}\mathrm{total}\mathrm{blood}\mathrm{loss}\;(\mathrm{mL})=\mathrm{BV}\times \frac{\mathrm{Preoperative}\mathrm{Hgb}-|\mathrm{POD}|3|\mathrm{days}|\mathrm{Hgb}}{\mathrm{Preoperative}\mathrm{Hgb}}\\ -\mathrm{Transfusion}\mathrm{amount}\;(mL) \end{array}$$

Notably, this retrospective study focused solely on radiographic healing assessed by mRUST scores. Patient-reported functional outcomes, such as pain scores, mobility assessments, or validated scales like the Harris Hip Score, were not systematically collected during the study period for all patients due to the retrospective nature of the data collection and oversights in prior clinical follow-up. This absence represents a limitation, as the full clinical impact and patient-perceived benefits of the observed differences in radiographic healing could not be comprehensively determined.

### 2.4. Statistical Analysis

Given the nature of the data and the small sample size, the Shapiro–Wilk test was performed to assess the normality of continuous variables. As most variables did not follow a normal distribution, non-parametric tests were chosen for comparisons. To minimize bias, mRUST scoring was performed independently and in a blinded manner by three orthopedic surgeons with no knowledge of treatment allocation. For consistency, the final score at each time point was defined as the median of the three raters’ assessments. Given the limited visualization of the medial cortex due to the nail, only lateral cortex scoring was used, resulting in a modified total score range of 3 to 12 instead of the conventional 4 to 16. While this modification was necessary due to intraoperative factors, it implies that direct quantitative comparisons with studies utilizing the full 16-point mRUST score should be made with caution. The exclusion of medial cortex assessment may also potentially overlook subtle aspects of medial side healing, although the lateral cortex is often considered critical for initial stability and load bearing in these fractures.

Nonunion was defined as incomplete healing by 12 months, accompanied by no radiographic evidence of progressive healing over a continuous 6-month period. Radiographic criteria for nonunion included persistent fracture lines, absence of bridging trabeculae, fracture edge sclerosis, and a lack of callus formation or remodeling [22].

All statistical analyses were performed using SPSS software version 25.0 (IBM Corp., Armonk, NY, USA). Continuous variables were reported as medians with interquartile ranges (Q1–Q3) and compared using the Mann–Whitney U test. Categorical variables were analyzed using Fisher’s exact test. A *p*-value < 0.05 was considered statistically significant, and 95% confidence intervals (CIs) were calculated where appropriate.

Given the small sample size and non-normal data distribution, the Mann–Whitney U test was selected for intergroup comparisons. This non-parametric test does not assume normality and is well suited for small samples and ordinal data. Similar applications in small-sample orthopedic studies have supported its validity and robustness [23,24,25].

It is acknowledged that the extremely small sample size (*n* = 5 vs. *n* = 7) is a major limitation of this study, inherently restricting statistical power. While a post hoc power analysis could provide insights into the likelihood of detecting true effects, its retrospective nature and the preliminary design of this study led us to prioritize direct presentation of observed differences. The chosen non-parametric Mann–Whitney U test is robust for small samples and non-normal data, and the consistency of significantly higher mRUST scores across multiple time points in the Plate + IM group suggests a consistent trend, despite the limited power to detect smaller effects or subtle differences in other parameters.

## 3. Results

### 3.1. Baseline Patient and Fracture Characteristics

Baseline demographic and perioperative parameters were compared between the IM and Plate + IM groups using the Mann–Whitney U test. No statistically significant differences were found in age, weight, height, BMI, hip and spinal BMD, lateral and medial cortical gaps, nail diameter, or canal–nail diameter difference (all *p* > 0.05), confirming the comparability of the two groups at baseline (Table 1 and Table 2).

### 3.2. Fracture Healing

**Figure 4:**
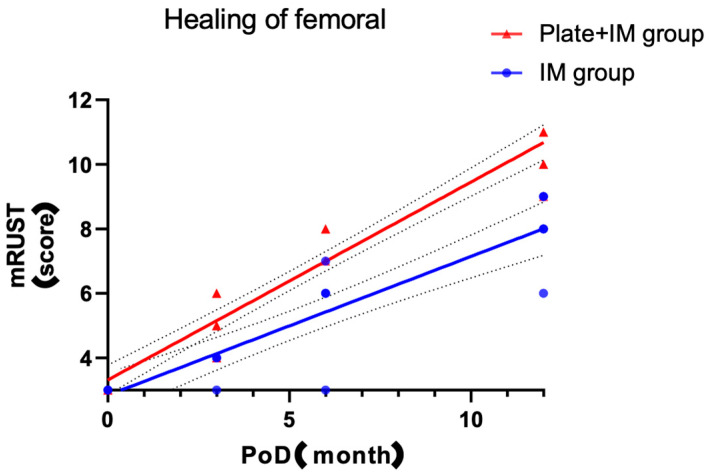
Comparison of radiographic fracture healing progress between the Plate + IM and IM groups. The graph illustrates modified Radiographic Union Score for Tibial fracture (mRUST) scores over 12 months post-surgery. The X-axis represents postoperative time in months; the Y-axis represents the mRUST score (range 3–12 due to exclusion of medial cortex scoring as detailed in Methods). Red triangles and line depict the Plate + IM group; blue circles and line depict the IM group. Both groups showed progressive increases in mRUST scores over time. The Plate + IM group demonstrated a visually steeper trajectory of mRUST progression. However, statistical interpretation of this trend is limited by the small number of timepoints.

**Figure 5:**
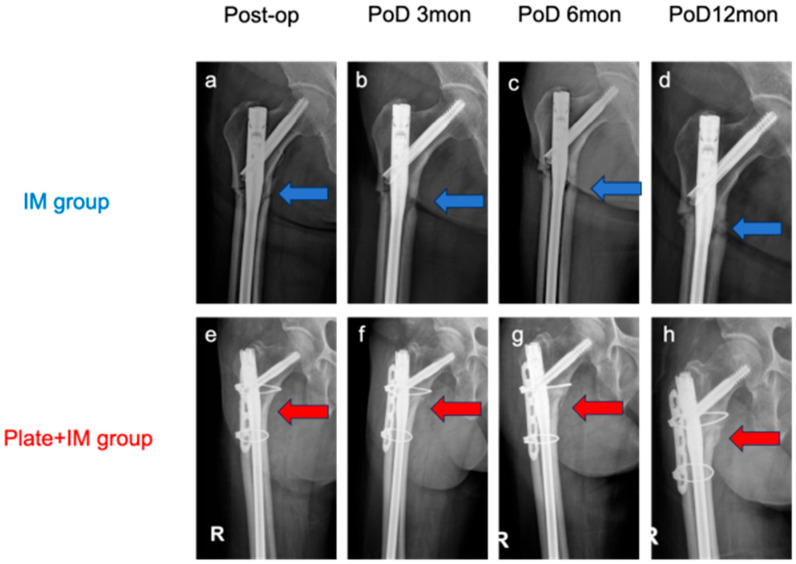
Serial postoperative radiographs illustrating fracture healing in representative cases from the IM group (**a**–**d**) and Plate + IM group (**e**–**h**). Radiographs were taken immediately after surgery (0 months) and at 3, 6, and 12 months. In the IM group case (**a**–**d**), blue arrows indicate the fracture site, where minimal callus formation is visible even at 12 months (**d**). In the Plate + IM group case (**e**–**h**), red arrows highlight the fracture site, showing progressive callus formation that becomes more evident by 12 months (**h**), suggesting more advanced radiographic healing.

Fracture healing, assessed using the mRUST score, was the primary outcome. The Plate + IM group demonstrated consistently higher mRUST scores at all postoperative time points. At 3 months, the median mRUST score was 5 (IQR 4–6) in the Plate + IM group versus 4 (IQR 3.5–4) in the IM group (*p* = 0.018). At 6 months, scores were 8 (IQR 8–8) and 6 (IQR 4.5–6.5), respectively (*p* = 0.003). At 12 months, scores reached 10 (IQR 10–11) versus 8 (IQR 7–9), respectively (*p* = 0.006). These significantly higher mRUST scores in the Plate + IM group correlated with the absence of nonunion cases (0%) in this group, compared to one nonunion (20%) observed in the IM group by 12 months post operation, as further detailed in the Complications section (Section 3.4). While a precise ‘time to union’ for all patients was not uniformly assessed due to the retrospective nature and varying follow-up periods, the consistently higher mRUST scores and the complete union in the Plate + IM group suggest a potentially accelerated radiographic healing trajectory (Table 2, Figure 4 and Figure 5).

While the Plate + IM group visually demonstrated a steeper upward trend in mRUST score progression, suggesting accelerated radiographic healing, it is important to note that interpreting a precise ‘slope’ based on only three distinct data points (3, 6, and 12 months) per group is statistically limited and should be approached with caution. The visual trend primarily highlights the consistently higher mRUST scores in the Plate + IM group across all time points (Table 3).

### 3.3. Perioperative Surgical Characteristics

Perioperative parameters were also compared. The median operative time was 160 min (IQR 130–215) in the Plate + IM group compared to 125 min (IQR 85–145) in the IM group; this difference was not statistically significant (*p* = 0.074). Estimated blood loss, calculated as described in the Methods section, was also comparable between groups: 643.8 mL (IQR 461.8–713.2) in the Plate + IM group and 593.4 mL (IQR 578.4–697.3) in the IM group (*p* = 0.685) (Table 3).

### 3.4. Complications

No major complications such as wound infection, implant loosening or breakage, or deep vein thrombosis were observed in either group. In the IM group, one case of fracture nonunion (20% of this cohort) was observed. This specific case, occurring in an elderly female patient, was characterized by a persistent fracture line on radiographs at three years postoperatively, accompanied by a complete absence of bridging callus formation and evidence of fracture edge sclerosis. Despite the long cephalomedullary nail remaining intact, there was no radiographic evidence of progressive healing over a continuous 6-month period, fulfilling our predefined criteria for nonunion. No cases of nonunion were observed in the Plate + IM group. The overall rates of reported complications did not differ significantly between the two groups (*p* = 0.417, Fisher’s exact test) (Table 4).

## 4. Discussion

This retrospective study evaluated the impact on the radiographic healing and key treatment-related outcomes of combining a lateral plate with a long intramedullary (IM) nail for the treatment of atypical subtrochanteric femoral fractures (ASFs). The results demonstrated significantly higher mRUST scores at 3, 6, and 12 months postoperatively in the Plate + IM group compared to the IM-only group. Notably, no cases of delayed union or nonunion were observed in the Plate + IM group, which, while not statistically significant given the sample size, clinically suggests a potential benefit of this combined approach for improved radiographic healing and earlier radiographic outcomes in patients with ASFs. While our study demonstrated significant differences in radiographic healing, it is crucial to acknowledge that this assessment relied solely on radiographic mRUST scores. The absence of patient-reported functional outcomes represents a significant limitation in fully understanding the clinical impact and patient-perceived benefits of these findings.

ASFs typically occur in elderly osteoporotic women, a population that presents substantial challenges for fracture fixation due to compromised bone quality and diminished healing potential. Standard IM nail fixation is often insufficient in this context because of anatomical and biomechanical factors specific to the subtrochanteric region. The proximal femoral canal is wider than the diaphyseal isthmus, limiting nail-to-cortex contact and reducing construct stability. Additionally, the subtrochanteric area is subjected to high biomechanical stress—tensile forces laterally and compressive forces medially—predisposing it to nonunion. Prior studies have reported nonunion rates of up to 20% with IM nailing alone [9,10,11].

Despite this clinical observation of no nonunion in the Plate + IM group versus one in the IM group, it is crucial to acknowledge that the small sample size of this study (*n* = 5 vs. *n* = 7) precludes drawing statistically significant conclusions regarding differences in nonunion rates between the two groups. This finding, therefore, remains suggestive and warrants investigation in larger and adequately powered studies.

In the present study, the addition of a lateral plate provided crucial supplemental fixation along the anterolateral femoral surface. This dual-fixation strategy, combining the intramedullary nail with a lateral plate, offers a biomechanically superior construct compared to IM nailing alone. While the intramedullary nail primarily resists bending and axial loads, the lateral plate acts as an effective buttress, directly counteracting the significant lateral tensile forces and resisting varus collapse, which are particularly prevalent and problematic in the subtrochanteric region. This synergistic effect enhances the overall rigidity and torsional stability of the construct, providing a more stable environment for fracture healing. The Plate + IM group consistently demonstrated superior mRUST scores across all time points, supporting the premise that this augmented stability contributes to more favorable radiographic healing. These results are supported by both established biomechanical principles and prior clinical observations. Despite the small sample size, the observed intergroup differences appear to reflect consistent trends, given the use of an appropriate non-parametric test and consistency across time points. These results are supported by both biomechanical principles and prior clinical observations. Despite the small sample size, the observed intergroup differences appear to reflect consistent trends, given the use of an appropriate non-parametric test and consistency across time points. Yang et al. emphasized the limitations of fixation in non-isthmal femoral regions, where IM nail stability is inherently weak [26]. The plate may function as a lateral buttress, resisting varus collapse and counteracting tensile forces, thereby improving alignment and load distribution across the fracture site [27,28].

Unlike conventional locking screws, which may be less reliable in osteoporotic bone, this study employed Dall–Miles cable fixation to secure the plate. This technique allowed the plate to conform closely to the femoral surface without excessive periosteal stripping, preserving local blood supply and potentially supporting biological healing. Previous studies have reported favorable outcomes with this method, though further investigation is needed to confirm its mechanical and biological advantages [29,30].

Regarding perioperative outcomes, no significant differences were observed in operative time or estimated blood loss between the two groups. Although dual fixation would intuitively increase operative complexity, the Plate + IM approach facilitated open reduction, simplifying fracture alignment and potentially offsetting the time added by plate application. Interestingly, the use of preliminary plate and cable fixation may have stabilized the fracture early in the procedure, thus facilitating IM nail insertion. As a result, this technique did not significantly prolong surgery or increase intraoperative bleeding. In contrast, the IM-only group often required traction-based reduction under C-arm guidance, which was technically demanding due to muscle tension (e.g., iliopsoas, gluteus medius) and a lack of reliable landmarks in transverse fracture patterns.

The findings of this study, suggesting accelerated radiographic healing in the Plate + IM group without a concomitant significant increase in operative time or estimated blood loss, carry potentially important clinical implications. For patients with ASFs—who are often elderly, present with osteoporotic bone, and are inherently at high risk for fixation failure and nonunion—a surgical strategy that could potentially promote more reliable and potentially faster bone consolidation is highly desirable. Such an approach could translate to earlier pain alleviation, facilitate expedited rehabilitation protocols, and ultimately reduce the likelihood of requiring secondary surgical interventions for delayed union or nonunion. Although this study focused on radiographic surrogate markers rather than direct functional outcomes, an improved healing trajectory may correlate with enhanced patient comfort and an earlier return to mobility, aspects of particular significance in this vulnerable patient cohort. The augmented biomechanical stability afforded by the dual-fixation construct may be especially advantageous in neutralizing the complex high-stress forces characteristic of the subtrochanteric femoral region, thereby contributing to a more favorable healing environment. Consequently, for patients with high-risk features for nonunion, such as severe osteoporosis, significant comminution, or large fracture gaps, this primary dual-fixation strategy could be considered as a potentially advantageous initial treatment option.

This study has several key limitations. Firstly, its retrospective design and small sample size (*n* = 12) inherently restrict statistical power, limit the generalizability of our findings, and precluded multivariate analysis; the results should, therefore, be considered exploratory. Secondly, patient allocation to treatment groups was non-randomized, primarily reflecting the introduction and increasing adoption of adjunctive plate augmentation in our department from approximately January 2016. While key baseline characteristics were comparable between the groups, this temporally influenced allocation strategy carries a potential for selection bias and the influence of unmeasured confounders (such as an evolution in perioperative care or surgeon experience over time) that cannot be entirely excluded. Specifically, potential factors known to influence fracture healing in ASFs, such as patterns of bisphosphonate discontinuation or the presence and severity of certain comorbidities, were not uniformly accounted for or analyzed in detail in this retrospective cohort, representing additional limitations. Thirdly, our assessment of fracture healing relied solely on radiographic mRUST scores. The absence of patient-reported functional outcomes means that the full clinical impact and patient-perceived benefits of the observed differences in radiographic healing could not be comprehensively determined. Finally, the inherent rarity of atypical subtrochanteric femoral fractures presents challenges for conducting large-scale prospective investigations. Consequently, these preliminary findings, while promising, warrant rigorous validation through future well-designed studies. Future research should prioritize prospective, multicenter, and randomized controlled trials with significantly larger cohorts to overcome the limitations of the current study.

Such trials should incorporate comprehensive functional assessments alongside radiographic evaluation, including validated patient-reported outcome measures and detailed evaluations of time to full weight-bearing and return to pre-injury activity levels. Furthermore, future studies should systematically account for and analyze the influence of potential confounding variables, such as specific bisphosphonate regimens, duration of use, discontinuation patterns, and a wider range of patient comorbidities, to provide a more robust understanding of the factors influencing healing in ASFs. Long-term follow-up is also crucial to assess the durability of fixation and the incidence of late complications.

## 5. Conclusions

The findings of this retrospective study suggest that combining a lateral plate with intramedullary nailing may enhance radiographic healing in atypical subtrochanteric femoral fractures without demonstrably increasing surgical trauma. The Plate + IM approach was associated with significantly higher mRUST scores at all postoperative time points, indicating improved fracture union. However, it is crucial to reiterate the significant limitations of this study, including its retrospective design, extremely small sample size (which rendered it underpowered to assess differences in nonunion rates or other complications), and reliance solely on radiographic outcomes. Given these limitations, this primary dual-fixation strategy may, therefore, offer a biomechanically advantageous alternative to IM nailing alone, particularly for osteoporotic patients at an increased risk of delayed union, but warrants further confirmation. Future prospective multicenter studies with larger cohorts are warranted to validate these promising radiographic results and, crucially, to evaluate long-term functional outcomes.

## Figures and Tables

**Table 1:** Comparison of data between the two groups of patients before surgery.

	IM Group (*n* = 5)	Plate + IM Group (*n* = 7)	*p*-Value
Age (years)	68.0 (64.0~79.0)	63.0 (62.0~67.0)	0.121
Weight (kg)	53.7 (51.0~56.5)	54.0 (51.3~57.0)	0.871
Height (cm)	158.8 (152.5~162.0)	155.0 (150.0~156.0)	0.167
BMI (kg/m^2^)	21.3 (20.22~23.445)	22.19 (21.56~25.33)	0.088
Hip BMD	0.899 (0.821~0.924)	0.840 (0.655~0.934)	0.684
Spinal BMD	1.032 (0.986~1.175)	0.984 (0.865~1.12)	0.223
Lateral Fracture Gap (cm)	1.9 (1.35~2.05)	1.0 (0.3~1.7)	0.167
Medial Fracture Gap (cm)	0.8 (0.75~2.15)	1.2 (0.4~2.5)	0.935

**Table 2:** Perioperative data.

	IM Group (*n* = 5)	Plate + IM Group (*n* = 7)	*p*-Value
Pre-Hgb (g/dL)	10.7 (10.25~11.45)	10.6 (10.1~10.9)	0.514
Post-Hgb (g/dL)	8.1 (7.7~8.8)	8.6 (7.7~8.8)	0.464
Hgb difference (g/dL)	2.8 (2.1~3.0)	1.9 (1.3~2.3)	0.073
Blood loss (mL)	593.4 (577.2~697.3)	643.8 (461.8~713.2)	0.685
OP time (min)	160 (130~215)	125 (85~145)	0.074
Diameter difference (mm)	2.2 (1.4–4.7)	1.0 (0.55–1.82)	0.061
Nail diameter (mm)	11 (10~12)	11 (11~12)	0.604

Diameter difference = femoral medullary canal diameter − intramedullary nail diameter. OP time, operation time.

**Table 3:** mRUST (healing score) of patients in the two groups at 3, 6, and 12 months after femoral fracture surgery.

mRUST	IM Group (*n* = 5)	Plate + IM Group (*n* = 7)	*p*-Value
3 months post-op	4 (3.5~4)	5 (4~6)	0.018
6 months post-op	6 (4.5~6.5)	8 (8~8)	0.003
12 months post-op	8 (7~9)	10 (10~11)	0.006

**Table 4:** Complications.

	IM Group (*n* = 5)	Plate + IM Group (*n* = 7)	*p*-Value
Infection	0	0	-
Implant Failure	0	0	-
Plate or Nail Breakage	0	0	-
Nonunion	1	0	0.417
Deep Vein Thrombosis (DVT)	0	0	-

## Data Availability

The datasets generated and analyzed during the current study are not publicly available due to patient privacy concerns but are available from the corresponding author upon reasonable request.

## Abbreviations

The following abbreviations are used in this manuscript:

ASF	Atypical Subtrochanteric Femoral Fracture
IM	Intramedullary
CMN	Cephalomedullary Nail
LCP	Locking Compression Plate
mRUST	Modified Radiographic Union Score for Tibial Fractures
BMD	Bone Mineral Density
BMI	Body Mass Index
POD	Postoperative Date
OP	Operation
BV	Blood Volume
DVT	Deep Vein Thrombosis
IQR	Interquartile Range
SPSS	Statistical Package for the Social Sciences
CI	Confidence Interval
